# Routine delirium monitoring in a UK critical care unit

**DOI:** 10.1186/cc7714

**Published:** 2009-02-09

**Authors:** Valerie J Page, Sachin Navarange, Sibu Gama, Danny F McAuley

**Affiliations:** 1 Department of Anaesthetics, Watford General Hospital, Vicarage Road, Watford, WD19 4DZ, UK; 2 Department of Anaesthetics, Inkosi Albert Luthuli Central Hospital, Private Bag X03, Mayville, 4038, South Africa; 3 Intensive Care Society Co-Director of Research, Centre for Infection and Immunity, School of Medicine, Dentistry and Biomedical Sciences, The Queen's University of Belfast, Grosvenor Road, Belfast, BT12 6BN, Northern Ireland, UK

## Abstract

**Introduction:**

Delirium in the intensive care unit (ICU) is associated with increased morbidity and mortality. Using an assessment tool has been shown to improve the ability of clinicians in the ICU to detect delirium. The confusion assessment method for the ICU (CAM-ICU) is a validated delirium-screening tool for critically ill intubated patients. The aim of this project was to establish the feasibility of routine delirium screening using the CAM-ICU and to identify the incidence of delirium in a UK critical care unit.

**Methods:**

Routine CAM-ICU monitoring was implemented in a mixed critical care unit in January 2007 following a two-month educational and promotional campaign. Guidelines for the management of delirium were introduced. During a two-month prospective audit in September and October 2007, the daily CAM-ICU was recorded by the bedside nurse for consecutive level 2 and level 3 patients admitted to the mixed medical/surgical critical care ward in a district general hospital. This was repeated in January 2008. Patient outcome was recorded. The records of an additional cohort of ventilated patients were reviewed retrospectively to determine compliance with routine CAM-ICU assessments.

**Results:**

Seventy-one patients were included in the observational cohort, with 60 patients in the retrospective cohort. In the prospective group it was not possible to assess for delirium with the CAM-ICU in nine patients due to persistent coma or inability to understand simple instructions. Excluding elective post-operative patients, the incidence of delirium was 45% in patients who could be assessed; in the 27 ventilated patients who could be assessed it was 63%. From the retrospective data compliance with the CAM-ICU assessment was 92%. The incidence of delirium in this retrospective group of ventilated patients who could be assessed was 65%.

**Conclusions:**

We have demonstrated that delirium screening is feasible in a UK ICU population. The high incidence of delirium and the impact on outcomes in this UK cohort of patients is in line with previous reports.

## Introduction

Delirium is an acute confusional state representing a serious and common clinical syndrome. It is a manifestation of acute brain dysfunction and is considered by some as a medical emergency [1]. To diagnose delirium requires that a patient have inattention with a disturbance in consciousness that developed over a short period of time, and which is caused by the direct physiological consequences of a general medical condition [2]. Delirium in the intensive care unit (ICU) is common with a reported incidence of up to 82% in ventilated patients using the confusion assessment method for the ICU (CAM-ICU) [3]. In the critically ill patient, the aetiology is often multifactorial [4].

In intensive care, even after adjusting for all confounding factors such as severity of illness and co-morbidities, delirium is an independent predictor of a three-fold increase in mortality at six months, as well as a three-fold higher intubation rate and more than 10 additional days in hospital [3,5,6]. Importantly delirium is associated with the subsequent development of dementia [7]. For patients with pre-existing cognitive impairment, a well-established predisposing risk factor, there may be a dramatic worsening of function.

UK as well as international guidelines recommends that delirium assessment should be part of routine ICU management [8,9]. The majority of delirium in critical care is either mixed or hypoactive, the motoric hypoactive subtype being more common in the elderly, characterised by psychomotor slowness and lethargy [10,11]. Evidence shows that delirium goes unrecognised in up to 66% of patients by nursing and medical staff [12]. Using a validated delirium assessment tool has been shown to improve the ability of physicians to detect delirium in ICU patients [13]. There are two validated tools to screen for delirium in intubated critical care patients, the CAM-ICU and the Intensive Care Delirium Screening Checklist (ICDSC) [14,15]. Both are based on *Diagnostic and Statistical Manual of Mental Disorders*, 4th Edition (*DSM *IV), criteria and the features of delirium. The CAM-ICU screens for four features at a single point in time and the ICDSC uses a screening checklist of eight features over the period of a nursing shift.

In the CAM-ICU, the patient is initially assessed for altered or fluctuating mental status, as well as inattention tested using a 10 letter sequence where the patient is required to squeeze the clinician's hand only when the letter A is stated. The patient is then assessed for disorganised thinking by their ability to answer four simple yes/no questions and a command, and finally for reduced level of consciousness. Patients are defined as delirious if altered mental status and inattention are present with disorganised thinking and/or reduced level of consciousness. This test can be performed on any patient who will open their eyes and keep their eyes open to a verbal stimulus, usually saying their name. Despite recommendations, routine delirium assessment is not undertaken as part of standard care in the UK. There are no published reports of delirium monitoring in a UK critical care unit using the CAM-ICU assessment tool. The aim of this study is to describe the use of the CAM-ICU and to determine the incidence and outcome of patients with delirium in a general critical care unit in a UK district general hospital.

## Materials and methods

This project was undertaken in Watford General Hospital which is a district and general hospital with an eight-bed mixed medical and surgical critical care unit. Routine CAM-ICU scoring for delirium once every 12-hour shift by the bedside nurse was introduced in January 2007 following a two-month educational programme of presentations, one-to-one teaching, posters and notices. Guidelines for the management of delirium were introduced.

Research ethics committee approval and informed consent was not required as the project was categorised as clinical audit by the Trust Research and Development Department as patient management was not altered, only routinely collected data were used and the data were fully anonymised.

During a two-month period in September and October 2007 and for a further one-month period in January 2008, data was prospectively collected on all consecutive admissions to the critical care unit. Subjects were a mixture of high dependency unit and ICU patients requiring differing levels of organ support or invasive monitoring. The CAM-ICU, as assessed by the bedside nurse, was recorded for their ICU stay. Patients were excluded from the final analysis if the nurse was unable to assess for delirium using the CAM-ICU at any time during the admission, either because of persistent coma – drug induced or as a result of their medical condition – or communication barriers. Patients were defined as having delirium if they fulfilled the diagnostic criteria according to the CAM-ICU on any assessment during their admission, that is they were defined as CAM-ICU positive. Patient demographics including acute physiology and chronic health evaluation (APACHE) II score were recorded. The patient's final outcome; death, discharge to a nursing home or discharge home was collected.

The records of an additional cohort of ventilated patients were reviewed retrospectively to determine ongoing compliance with daily routine CAM-ICU assessments in November and December 2007 and February and March 2008. If the CAM-ICU assessment was not recorded on a 24-hour chart this was documented as non-compliance.

### Statistical analysis

Proportions were used as descriptive statistics for categorical variables and median (interquartile range) for continuous variables. 95% confidence intervals (CI) for hospital mortality in each group were calculated using the *t *distribution.

## Results

Delirium incidence data were collected on 80 patients prospectively, from which nine patients were excluded from the final analysis as they were unable to be screened for delirium using the CAM-ICU throughout their admission. Reasons that patients could not be assessed were that they remained in a coma throughout the admission (n = 7), had learning disabilities (n = 1) or did not understand English (n = 1). Patient demographics are summarised in Table 1; APACHE II scores for the first 24 hours were available for 52 of the patients, 21 post-operative patients and 31 emergency admissions. Twenty-three of the 71 patients were elective post-operative patients, 22 of whom were never delirious while in the ICU. When elective post-operative patients were excluded, the overall incidence of delirium was 45%. Thirty-four patients in the prospective audit were ventilated (Table 2). Twenty-seven of those could be assessed. In this subgroup, delirium incidence was 63% (Table 3). In the retrospective cohort of 60 ventilated patients, the compliance with recording the daily CAM-ICU assessment was over 92% (Table 4). The incidence of delirium, that is those patients who screened positive for delirium at least once during admission, in the 48 patients in this group who could be assessed was 65%.

**Table 1:** Demographic data for 71 patients admitted consecutively and screened prospectively for delirium

	Delirious	Not delirious
Number of patients (%)	22 (31%)	49 (69%)

Age, years	70 (56 to 76)	73 (60 to 77)

Male (%)	16 (72%)	34 (69%)

APACHE II	21 (17 to 30)	15 (11 to 20)

Deaths	8	5

Mortality (95% confidence interval)	36% (15 to 57%)	8% (3 to 16%)

Data are n (%) or median (interquartile range).

APACHE = acute physiology and chronic health evaluation.

**Table 2:** Incidence of delirium according to patient subgroups

	Delirious	Not delirious
Elective post-operative (n = 23)	1 (4%)	22 (96%)

Emergency admissions (n = 57)	22 (45%)	27 (55%)

Ventilated patients (n = 27)	17 (63%)	10 (37%)

**Table 3:** Demographic data for subgroup of ventilated patients able to be screened for delirium (n = 27)

	Delirious	Not delirious
Number of patients (%)	17 (63%)	10 (37%)

Age, years	70 (56 to 76)	72 (45 to 76)

Male	12 (50%)	5 (50%)

APACHE II	23 (18 to 34)	19 (9 to 35)

Deaths	4	1

Mortality	24%	10%

Data are n (%) or median (interquartile range).

APACHE = acute physiology and chronic health evaluation.

**Table 4:** Daily CAM-ICU documented on nursing charts reviewed retrospectively in 60 ventilated patients

CAM-ICU recorded, compliance* (%)	726 (92%)
Positive	156

Negative	374

UTA	196

CAM-ICU not recorded, non-compliance	60 (8%)

*Compliance was defined as one CAM-ICU assessment during each 24-hour period. If the CAM-ICU was positive at any screening on a daily chart this was recorded as CAM-ICU positive for that day.

CAM-ICU = confusion assessment method for intensive care units; UTA = unable to assess.

## Discussion

Although delirium is more common in sicker patients, it is independently associated with worse outcomes after adjusting for common confounding factors including severity of illness [3]. In the US, delirium is associated with 39% higher ICU and 31% higher hospital costs [16]. Despite this, there has been reluctance in the UK to implement routine delirium monitoring [17]. Reasons quoted for this include: a lack of familiarity with the assessment tools that are perceived to be designed for research rather than use in clinical practice; a perception of limited clinical use outside the validating centres and in specific patient groups; a lack of clarity as to who was responsible for assessing delirium; clinician time constraints; and finally the belief that the highly sedated, and by implication sicker, patients, cannot be screened.

The CAM-ICU was developed as a brief, accurate and reliable instrument for use by nurses and physicians to identify delirium in ICU patients [14]. The bedside nurse is, in the majority of units, the most appropriate clinician to screen patients for delirium. The CAM-ICU is easy to administer, takes on average less than one minute to complete and requires minimal training. In the CAM-ICU validation study conducted by Ely and colleagues the average APACHE II score was 25.6 in delirious ventilated patients demonstrating CAM-ICU scoring is feasible in a severely ill patient cohort [14].

Prior to implementing delirium screening the consultant intensivists on the ICU reviewed the two validated screening tools available and made a pragmatic decision to use the CAM-ICU. The alternative to the CAM-ICU is the ICDSC. Although they both demonstrate high sensitivity scores of 99%, the ICDSC has a specificity of 64% as compared with the CAM-ICU of 96% [14,15].

We have demonstrated that the use of CAM-ICU to monitor delirium is feasible and is performed routinely in all patients at least once a day over 92% of the time. This level of compliance with delirium screening, once nurses are familiar with the tool, was also seen in a large-scale implementation programme of delirium monitoring in two medical centres in the US, one a University hospital and the other a community Veterans' hospital where nurses checked the CAM-ICU more than once in 63% of shifts [18]. If delirium is hypoactive it is likely to go unrecognised unless a screening tool is used.

The high incidence of delirium in both our prospective and retrospective cohorts in this UK population is in line with previous reports from other countries studying a similar case mix (Table 5).

**Table 5:** Incidence of delirium in the critically ill using CAM-ICU, available published data since 2005

Country	N	Case mix	APACHE II	Incidence ICU
USA 2008 [30]	336	Ventilated > 12 hours MICU	26*	71 to 74% 28 days

Germany 2008 [31]	174	SICU including elective % vent not available	25**	41%

USA 2007 [32]	304	MICU 54% ventilated	20 to 25**	70% within 48 hrs

Sweden 2007 [33]	14	Ventilated	19**	48%

USA 2006 [11]	614	MICU 49% ventilated	20**	72% in 65 years or older 57% younger than 65 years

USA 2005 [34]	93	MICU ventilated	21*	47%

Acute physiology and chronic health evaluation (APACHE) II *median **mean

CAM-ICU = confusion assessment method for intensive care unit; ICU = intensive care unit; MICU = medical intensive care unit; SICU = surgical intensive care unit.

Of note, of the patients who were excluded, nine were unable to be assessed, seven because they were comatose throughout the stay, either due to severe brain injury or due to sedation levels, one did not speak English and one had significant learning disabilities. Eight of these patients died. Coma in critical illness is associated with a high risk of developing delirium and the study by Ely and colleagues also demonstrated that patients who spent time in a coma had worse outcomes [3]. More data are needed on this group of patients, not only for mortality but cognitive outcomes.

Although this study was not powered to investigate the effect of delirium on mortality, in the patients studied prospectively, mortality in the delirium group was higher compared with the group that did not develop delirium. This is in keeping with data from a number of studies that have demonstrated an increased risk of mortality associated with delirium [3,5,6].

Given the incidence and adverse effect on outcomes associated with delirium, it has been suggested that patients be given prophylaxis or treated for delirium [19-21]. Any underlying cause of delirium such as infection should be identified and managed. It remains unproven whether treatment of delirium improves outcome. In elderly general medical patients, using a non-pharmacological 'delirium bundle' to prevent or reduce the duration of delirium has been shown to significantly improve outcomes in terms of hospital mortality and length of stay in three studies [22-24]. There are limited data to guide the administration of antipsychotic medication to delirious critically ill patients. Antipsychotics such as haloperidol are the most commonly used pharmacological treatment [25]. Haloperidol is recommended in national and international guidelines [8,9]. Milbrandt and colleagues demonstrated a decrease in mortality in mechanically ventilated patients in a retrospective cohort analysis [26]. However, in contrast, recent publications suggest the potential for increased morbidity and mortality from the use of antipsychotics in elderly patients [27]. There is, therefore, a rationale and urgent need for research into the management of delirium with haloperidol in a large multicentre placebo-controlled clinical trial powered for mortality [28,29].

## Conclusions

We have demonstrated that delirium screening is feasible and can be performed routinely in all patients over 92% of the time in a UK intensive care population. The high incidence of delirium and the impact on outcomes in this UK cohort of patients is in line with previous reports from other countries studying a similar case mix. There is, therefore, a rationale and urgent need to develop strategies to treat delirium in critically ill patients. In the meantime to measure is to know; so all UK critical care units should screen patients for delirium as a routine.

## Key messages

• Routine delirium screening is feasible in a UK ICU.

• In keeping with international data, delirium is common; the incidence of delirium in ventilated patients is 63%.

• Nurse compliance with routine daily screening for delirium is high.

## Abbreviations

APACHE: acute physiology and chronic health evaluation; CAM-ICU: confusion assessment method for intensive care units; CI: confidence interval; DSM-IV: *Diagnostic and Statistical Manual of Mental Disorders*, 4th Edition; ICDSC: intensive care delirium screening checklist; ICU: intensive care unit.

## Competing interests

The authors declare that they have no competing interests.

## Authors' contributions

VP conceived, designed the audit and contributed to the collection of data. SN and SG collected and collated data. VP wrote the initial manuscript draft. DMcA participated in the interpretation and analysis of the results, and reviewed the manuscript drafts and formulated the final manuscript. All authors read and approved the final manuscript.

## References

[B1] Caraceni A, Grassi L (2003). Delirium – acute confusional states in palliative medicine.

[B2] American Psychiatric Association (1994). Diagnostic and Statistical Manual of Mental Disorders.

[B3] Ely EW, Shintani A, Truman B, Speroff T, Gordon SM, Harrell FE, Inouye SK, Bernard GR, Dittus RS (2004). Delirium as a predictor of mortality in mechanically ventilated patients in the intensive care unit. JAMA.

[B4] Ely E, Gautam S, Margolin R, Francis J, May L, Speroff T, Truman B, Dittus R, Bernard G, Inouye S (2001). The impact of delirium in the intensive care unit on hospital length of stay. Intensive Care Med.

[B5] Lin S-M, Liu C-Y, Wang C-H, Lin H-C, Huang C-D, Huang P-Y, Fang Y-F, Shieh M-H, Kuo H-P (2004). The impact of delirium on the survival of mechanically ventilated patients. Crit Care Med.

[B6] Ouimet S, Kavanagh BP, Gottfried SB, Skrobik Y (2007). Incidence, risk factors and consequences of ICU delirium. Intensive Care Med.

[B7] Jackson J, Gordon S, Hart R, Hopkins RO, Ely EW (2004). The association between delirium and cognitive decline: a review of the empirical literature. Neuropsychol Rev.

[B8] Jacobi J, Fraser G, Coursin D, Riker R, Dorrie RN, Wittbrodt ET, Chalfin DB, Masica MF, Bjerke HS, Scott H, Coplin WM, Crippen DW, Fuchs BD, Kelleher RM, Marik PE, Nasraway SA, Murray MJ, Peruzzi WT, Lumb PD (2002). Clinical practice guidelines for the sustained use of sedatives and analgesics in the critically ill adult. Crit Care Med.

[B9] Willson J, Shah A, Page V, Manji M (2008). Intensive Care Society Guidelines for Sedation of Critical Care Patients.

[B10] Meagher D, O'Hanlon D, O'Mahoney E, Casey P, Trzepacz P (2000). Relationship between symptoms and motoric subtype of delirium. J Neuropsychiatry Clin Neurosci.

[B11] Peterson J, Pun B, Dittus R, Thomason J, Jackson J, Shintani A, Ely EW (2006). Delirium and its motoric subtypes: a study of 614 critically ill patients. J Am Geriatr Soc.

[B12] Inuoye SK, Foreman MD, Mion LC, Katz K, Cooney L (2001). Nurses' recognition of delirium and its symptoms: comparison of nurse and researcher ratings. Arch Intern Med.

[B13] Devlin J, Fong J, Schumaker G, O'Connor H, Ruthazer R, Garpestad E (2007). Use of a validated delirium assessment tool improves the ability of physicians to identify delirium in medical intensive care unit patients. Crit Care Med.

[B14] Ely EW, Margolin R, Francis J, May L, Truman B, Dittus R, Speroff T, Gautam S, Bernard G, Inouye S (2001). Evaluation of delirium in critically ill patients: Validation of the Confusion Assessment Method for the Intensive Care Unit (CAM-ICU). Crit Care Med.

[B15] Bergeron N, Dubois MJ, Dumont M, Dial S, Skrobik Y (2001). Intensive Care Delirium Screening Checklist: evaluation of a new screening tool. Intensive Care Med.

[B16] Milbrandt EB, Deppen S, Harrison PL, Shintani AK, Speroff T, Stiles RA, Truman B, Bernard GR, Dittus RS, Ely EW (2004). Costs associated with delirium in mechanically ventilated patients. Crit Care Med.

[B17] Devlin JW, Fong JJ, Fraser GL, Riker R (2007). Delirium assessment in the critically ill. Intensive Care Med.

[B18] Pun BT, Gordon SM, Peterson J, Shintani A, Jackson J, Foss J, Harding S, Bernard G, Dittus R, Ely EW (2005). Large-scale implementation of sedation and delirium monitoring in the intensive care unit; a report from two medical centers. Crit Care Med.

[B19] Yende S, Wunderink R (2002). Causes of prolonged mechanical ventilation after coronary artery bypass surgery. Chest.

[B20] American Psychiatric Association Practice guidelines Treatment of patients with delirium. http://www.psychiatryonline.com/pracGuide/pracGuideTopic_2.aspx.

[B21] Tanios M, Epstein SK, Teres D (2004). Are we ready to monitor for delirium in the intensive care unit?. Crit Care Med.

[B22] Lundstrom M, Edlund A, Karlsson S, Brannstrom B, Bucht G, Gustafson Y (2005). A multifactorial intervention program reduces the duration of delirium, length of hospitalization, and mortality in delirious patients. J Am Geriatr Soc.

[B23] Naughton B, Saltzman S, Ramadan F, Chadha N, Priore R, Mylotte J (2005). A multifactorial intervention to reduce prevalence of delirium and shorten hospital length of stay. J Am Geriatr Soc.

[B24] Inouye S, Bogardus S, Leo-Summers L, Acampora D, Holford T, Cooney L (1999). A multicomponent intervention to prevent delirium in hospitalized older patients. New Engl J Med.

[B25] Patel RP, Speroff T, Scott TA, Pandharipande P, Girard T, Dittus R, Bernard G, Ely EW (2009). Delirium and sedation in the Intensive care unit (ICU): survey of behaviours and attitudes of 1384 health care professionals. Crit Care Med.

[B26] Milbrandt EB, Kersten A, Kong L, Weissfeld L, Clermont G, Fink M, Angus D (2005). Haloperidol is associated with lower hospital mortality in mechanically ventilated patients. Critical Care Medicine.

[B27] Wang P, Schneeweiss S, Avorn J, Fischer M, Mogun H, Solomon D, Brookhart M (2005). Risk of death in elderly users of conventional vs. atypical antipsychotic medications. New Engl J Med.

[B28] Siddiqui N, Stockdale R, Britton AM, Holmes J (2007). Interventions for preventing delirium in hospitalised patients. Cochrane Database Syst Rev.

[B29] Ryan C (2001). Optimising management of delirium; placebo controlled trials of pharmacological treatments are needed. BMJ.

[B30] Girard T, Kress J, Fuchs B, Thomason J, Schweickert W, Pun B, Taichman D, Dunn J, Pohlman A, Kinniry P, Jackson J, Canonico A, Light R, Shintani A, Thompson J, Gordon S, Hall J, Dittus R, Bernard G, Ely EW (2008). Efficacy and safety of a paired sedation and ventilator weaning protocol for mechanically ventilated patients in intensive care (Awakening and Breathing Controlled trial): a randomized controlled trial. Lancet.

[B31] Plaschke K, von Haken R, Scholz M, Engelhardt R, Brobeil A, Martin E, Weigand M (2008). Comparison of the confusion assessment method for the ICU (CAM-ICU) with the Intensive Care Delirium Screening Checklist (ICDSC) for delirium in critical care patients gives high agreement rate(s). Intensive Care Med.

[B32] Pisani M, Murphy T, Ness P, Araujo K, Inouye S (2007). Characteristics associated with delirium in older patients in a medical intensive care unit. Arch Intern Med.

[B33] Larsson C, Granberg A, Ersson A (2007). Confusion assessment method for the intensive care unit (CAM-ICU): translation, retranslation and validation into Swedish intensive care settings. Acta Anaesthesiol Scand.

[B34] Micek S, Anand N, Laible B, Shannon W, Kollef M (2005). Delirium as detected by the CAM-ICU predicts restraint use among mechanically ventilated medical patients. Crit Care Med.

